# A mathematical model of the interaction of abscisic acid, ethylene and methyl jasmonate on stomatal closure in plants

**DOI:** 10.1371/journal.pone.0171065

**Published:** 2017-02-09

**Authors:** Allen Lamarca Nazareno, Bryan Sapon Hernandez

**Affiliations:** 1 Institute of Mathematical Sciences and Physics, University of the Philippines Los Baños, Laguna, Philippines; 2 College of Arts and Science, Malayan Colleges Laguna, Cabuyao, Laguna, Philippines; Universidade Federal de Vicosa, BRAZIL

## Abstract

Stomatal closure is affected by various stimuli such as light, atmospheric carbon dioxide concentration, humidity and phytohormones. Our research focuses on phytohormones, specifically: abscisic acid (ABA), ethylene (ET) and methyl jasmonate (MeJA) that are responsible for the regulation of several plant processes, especially in guard cell signalling. While several studies show that these three phytohormones cause stomatal closure in plants, only two studies are notable for establishing a mathematical model of guard cell signalling involving phytohormones. Those two studies employed Boolean modelling and mechanistic ordinary differential equations modelling. In this study, we propose a new mathematical model of guard cell transduction network for stomatal closure using continuous logical modelling framework. Results showed how the different components of the network function. Furthermore, the model verified the role of antioxidants in the closure mechanism, and the diminished closure level of stomata with combined ABA-ET stimulus. The analysis was extended to ABA-ET-MeJA crosstalk.

## Introduction

Stomata are microscopic pores commonly found in the lower epidermis of plant leaves that are very important in the growth and survival of plants [1]. Each pore is formed by two guard cells that regulate the stomatal closure mechanism by controlling turgor pressure on them. When the guard cells are swollen, the pore opens. In contrast, when the guard cells are flaccid, the pore closes [2]. The loss of turgor pressure is a consequence of the efflux of ions out of the guard cells which results to stomatal closure [3].

The opening and closing of the stomata is caused by a variety of stimuli such as light, atmospheric carbon dioxide concentration, humidity and plant hormones [2,4]. For instance, during hot or dry days, plants close their stomata as a natural response to conserve water [3]. It is important for the plants to regulate stomatal closure to adapt to these external challenges.

Drought stress is a major abiotic condition that has adverse effects on plant growth and yield. Water deficiency may result to cellular dehydration leading to damage and may eventually be fatal to plants [5]. As defense response to water stress, certain plant hormones trigger stomatal closure. One of these hormones is abscisic acid (ABA) which is synthesized during drought stress. As the soil dries, ABA builds up in leaves, thus promoting closure [2,6]. Ethylene (ET) is another effector of stomatal closure. It is involved in the regulation of various plant processes [7]. Although both ABA and ET are known to cause stomatal closure, they fail to achieve full closure when applied simultaneously [2]. Likewise, jasmonates are phytohormones that trigger closure. They regulate plant processes such as pollen maturation and tendril coiling. Methyl jasmonate (MeJA) is a volatile methyl ester of jasmonic acid which has been used in studying jasmonic signaling pathway [8–10].

Various studies have described crosstalk in guard cell signalling [1,2,8,9,11,12]. Some of these studies include the interaction between hormones such as ABA, ET and MeJA [1,2,8,9]. One notable work about guard cell signalling was the study of Li et al. [13] which adapted Boolean modelling in predicting essential components of the ABA guard cell signalling transduction network. Beguerisse-Diaz et al. [2], on the other hand, have developed an ordinary differential equation (ODE) model of stomatal closure based on biochemical pathway information. They were able to describe the role of antioxidant mechanisms in the lack of stomatal closure when guard cells are subjected to the combined stimulus of ABA and ET.

However, using ODEs to describe the biochemical processes of a system requires adequate information about biological mechanisms and kinetic parameters—which implies that mechanistic ODE modelling may not be possible. In this study, we explored on analyzing the guard cell signalling network using continuous logical modelling. This modelling formalism was developed by Mendoza and Xenarios [14] and was described as a semi-quantitative technique dealing with logic-based ordinary differential equations. Although it cannot provide comprehensive quantitative information about signalling network, it can confer substantial qualitative information such as trends in various biological networks. Moreover, analysis using semi-quantitative modelling can be useful in determining essential properties of the network through its topology despite the unavailability of experimental data [15]. Sankar et al. [16] have successfully used the continuous logical framework to analyze cellular auxin and brassinosteriod signalling and their interaction.

The interaction between the three phytohormones ABA, ET and MeJA in guard cell signalling was the focus of this study. To our knowledge, no mathematical models on stomatal closure have considered the interaction among these hormones.

This paper proceeds with a further discussion of signal transduction network of stomatal closure. We then describe the methodology used to construct the mathematical model of guard cell signalling pathway. Results and discussions highlight the role of antioxidants, the effect of the combined ABA-ET stimulus and the inclusion of MeJA in the network in the closure mechanism. Based on these, we draw conclusions on the guard cell signalling network.

## Signal transduction for stomatal closure

The phytohormones ABA, ET and MeJA are known to be effectors of stomatal closure in plants. An integrated ABA and ethylene signalling network in guard cells was shown in the study of [2]. Additionally, [9] proposed signalling pathway and signal crosstalk between MeJA and ABA in guard cells. Based on these two studies and supported by other relevant literature, we constructed a signal transduction network. It should be noted, however, that the model established in [2] was considered in this study with additional connections to incorporate MeJA in the network.

There are several identified components in the integrated ABA and ethylene signalling network in guard cells. Reactive oxygen species (ROS) and nitric oxide (NO) are central components of the signalling network that regulate stomatal movement in response to hormones such as ABA [7]. ABA sequesters the protein phosphatase 2C ABA-insensitive 1 (ABI1) which lead to phosphorylation of NADPH-oxidase *Arabidopsis thaliana* respiratory burst oxidase homolog F (AtrbohF) by kinase open stomata 1 that produces ROS such as superoxide and hydrogen peroxide [17]. During the ethylene-induced stomatal closure, AtrbohF upregulates the production of ROS [18]. Consequently, the increase on the level of ROS results to the increased production of NO through the nitrate reductase 1 (NIA1) [19–21].

Other ABA-induced cellular responses include the activation of vacuolar proton pumps that promote the cytosolic pH (pH_cyt_) [22]. The positive regulation of NO and pH_cyt_ reduces the concentration of K^+^ ions by increasing its efflux and decreasing the influx. An increased efflux means an escalation of the number of available outwards-rectifying K^+^ channels (I_K,out_), while reduced influx means a down-regulation of inwards-rectifying K^+^ channels(I_K,in_) [12,23,24]. The resulting lower concentration of K^+^ ions leads to the loss of turgor and eventually to the closure of the stomatal pore [4,23]. We denote $K_{out}^{+}$ as active I_K,out_ and $K_{in}^{+}$ as active I_K,in_.

Antioxidants are also essential components of the transduction network. Based on experimental data, the network in [2] suggests two antioxidant mechanisms which are active in guard cells. Two antioxidants included were described by AXO_1_ and AXO_2_ lying at the end of two linear activation cascades due to doses of ABA and ET. One is a generic antioxidant in response to an individual stimulus, ABA or ET. This mechanism allows ROS to signal downstream to control oxidative stress of around two hours. The other is an antioxidant response which is active only when both hormones, ABA and ET, are present simultaneously. This response happens for about 10 minutes, and disrupts the closure process. Additional hypothesized connections are then established, which include the activation of both pH_cyt_ and NO by ET.

**Fig 1:**
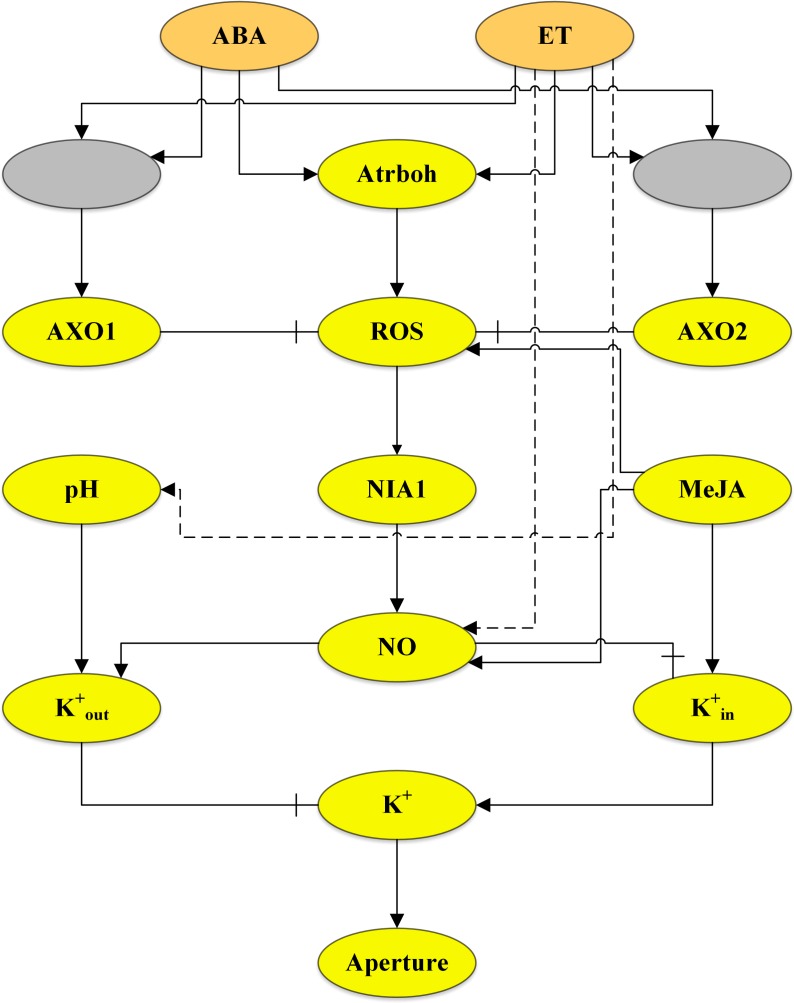
Integrated ABA, ethylene and MeJA signalling network in guard cells. The hormones ABA, ET, and MeJA are the input nodes shown as orange ellipses. The two grey ellipses represent components of the network that are not yet known and regarded as linear activation cascades. Yellow ellipses are the variable nodes. Interactions between the components are shown by the green lines connecting them. Activation or production is represented as solid lines ending in an arrowhead while inactivation, repression, or scavenging is represented by solid lines ending in a hammerhead. Broken lines represent hypothesized connections that require experimental validation.

ROS and NO play a significant role in MeJA-induced stomatal closure. It is shown that MeJA induces ROS in guard cells. Similarly, MeJA facilitates the production of NO to induce stomatal closure [9]. Fig 1 shows the complete transduction network in guard cells involving the three phytohormones.

## Methodology

### Construction of the different models of the guard cell

#### Signalling pathway

Sixteen models have been considered in the analysis of the guard cell signalling network. These models include all 16 components of the guard cell transduction system shown in Fig 1. The variations were made by removing connections from one component to the other and incorporating logical connections, delay, and other assumptions. Table 1 provides a summary of the models.

**Table 1:** Sixteen models of the guard cell signalling.

Model	C1	C2	C3	C4	C5	C6	C7
M1	Yes	No	No	No	No	No	No
M2	Yes	No	Yes	No	No	No	No
M3	Yes	Yes	No	No	No	No	No
M4	Yes	Yes	Yes	No	No	No	No
M5	Yes	Yes	No	Yes	No	No	No
M6	Yes	Yes	Yes	Yes	No	No	No
M7	Yes	Yes	No	No	Yes	No	No
M8	Yes	Yes	Yes	No	Yes	No	No
M9	Yes	Yes	No	No	Yes	Yes	No
M10	Yes	Yes	Yes	No	Yes	Yes	No
M11	Yes	Yes	No	No	Yes	No	Yes
M12	Yes	Yes	Yes	No	Yes	No	Yes
M13	Yes	Yes	No	No	Yes	Yes	Yes
M14	Yes	Yes	Yes	No	Yes	Yes	Yes
M15	Yes	Yes	No	No	No	No	Yes
M16	Yes	Yes	Yes	No	No	No	Yes

where

C1: Inclusion of the 16 components
C2: Logical *and* operation on ABA and ET signals to activate AXO_2_ without cascading
C3: Incorporation of cascading to activate AXO_1_ and AXO_2_
C4: Activation of AXO_1_ is only affected by ABA
C5: Activation of AXO_1_ is only affected by ET
C6: Elimination of the connection of ET to pH
C7: Elimination of the connection of ET to NO

#### Implementation of continuous logical modelling framework

**Fig 2:**
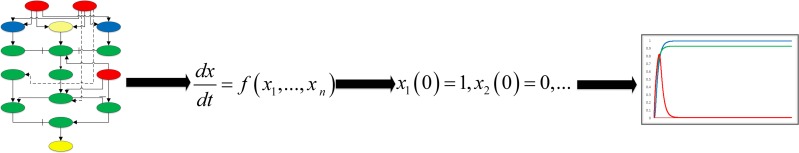
Schematic diagram of constructing a continuous dynamical system.

A continuous logical modelling was used in analyzing the guard cells transduction network. Mendoza and Xenarios [14] have proposed a standard technique of transforming a signalling network into a continuous dynamical system model. The first step in continuous logical modelling formalism is to convert the network into a continuous dynamical system. This is followed by setting the initial state of the variables and specifying all the parameter values. The system is then run until it converges to a steady state. The complete process is illustrated in Fig 2.

To describe the network as a continuous dynamical system, the following set of ordinary differential equations was used:

Let $\left\{x_{n}^{a}\right\}$ be the set of activators and $\left\{x_{m}^{i}\right\}$ be the set of inhibitors of *x*_*i*_, for every species *x*_*i*_. Then

$$\frac{dx_{i}}{dt}=\frac{-e^{0.5h}+e^{-h\left(\omega_{i}-0.5\right)}}{\left(1-e^{0.5h}\right)\left(1+e^{-h\left(\omega_{i}-0.5\right)}\right)}-\gamma_{i}x_{i}$$ (1)

$$\omega_{i}=\{\begin{array}{c} \left(\frac{1+\sum \alpha_{n}}{\sum \alpha_{n}}\right)\left(\frac{\sum \alpha_{n}x_{n}^{a}}{1+\sum \alpha_{n}x_{n}^{a}}\right)\left(1-\left(\frac{1+\sum \beta_{m}}{\sum \beta_{m}}\right)\left(\frac{\sum \beta_{m}x_{m}^{i}}{1+\sum \beta_{m}x_{m}^{i}}\right)\right)\;*\\ \left(\frac{1+\sum \alpha_{n}}{\sum \alpha_{n}}\right)\left(\frac{\sum \alpha_{n}x_{n}^{a}}{1+\sum \alpha_{n}x_{n}^{a}}\right)\;**\\ 1-\left(\frac{1+\sum \beta_{m}}{\sum \beta_{m}}\right)\left(\frac{\sum \beta_{m}x_{m}^{i}}{1+\sum \beta_{m}x_{m}^{i}}\right)\;*** \end{array}$$ (2)

where * indicates use if *x*_*i*_ has both activators and inhibitors, ** indicates use if *x*_*i*_ has activators only, and, *** indicates use if *x*_*i*_ has inhibitors only.

The differential equation’s right-hand side incorporates an activation function and a term for decay. The activation is a sigmoid function of *ω* which corresponds to the total input to the node. The values of the following variables and parameters were given as follows: 0 ≤ *x*_*i*_ ≤ 1, 0 ≤ *ω*_*i*_ ≤ 1, *h*, *α*_*n*_, *β*_*m*_ and *γ*_*i*_ > 0. Since the value of node *x* is bounded to be within the interval [0,1], it suggests that the level of activation is normalized and not an absolute value. The decay rate *γ*_*i*_ is directly proportional to the level of activation of the node [14]. The total input to the node is an incorporation of several inhibition and activation mechanisms acting on a node. The function *ω* is defined in three forms to account for the effect of both activatory and inhibitory information. The weight of the activators and inhibitors are represented by the parameters *α* and *β*, respectively. Moreover, *ω* gives a bounded sigmoid form, regardless of the values of *α* and *β*. The parameter *h* is called the gain of the sigmoid function and controls the steepness of the curve.

Each model constructed has a total of 13 differential equations associated with the mechanism of the signalling network. These equations are as follows:

$$\frac{dx_{i}}{dt}=\frac{-e^{0.5h}+e^{-h\left(\omega_{i}-0.5\right)}}{\left(1-e^{0.5h}\right)\left(1+e^{-h\left(\omega_{i}-0.5\right)}\right)}-\gamma_{i}x_{i},\;i=3,4,\ldots ,15$$ (3)

The values of the parameters *α*, *β* and *γ* are all set to 1, while parameter *h* = 10. These are default values adapted from the work of Mendoza and Xenarios [14]. A software package called Berkeley Madonna, created by Robert Macey and George Oster of the University of California at Berkeley [25], was used to solve the system of ODEs. The fixed delay mechanism and the logical *and* operation were incorporated using default functions in the software. The delay was added to incorporate the activation mechanism of the two antioxidants. The initial activation levels of the involved components were all set to zero, except for *x*_1_, *x*_2_ and *x*_16_ which are the input variables. The models were run using the different values of the input variables. The effect of the hormones on the various components of the network, upon introduction to the system, was observed. The simulation started with all input variables set to 0. The level was increased by increments of 0.1 until the maximum level of 1.0 was achieved. The response of the different components was then observed. Several combinations of these values for ABA, ET and MeJA levels were used for the simulation process. The list of notations used in the implementation is given in Table 2.

**Table 2:** Notations used in the continuous logical model.

Component	Notation
ABA	*x*_1_
ET	*x*_2_
Unknown component 1	*x*_3_
Atrboh	*x*_4_
Unknown component 2	*x*_5_
AXO_1_	*x*_6_
ROS	*x*_7_
AXO_2_	*x*_8_
pH	*x*_9_
NIA1	*x*_10_
NO	*x*_11_
${\text{K}}_{\text{out}}^{+}$	*x*_12_
${\text{K}}_{\text{in}}^{+}$	*x*_13_
K^+^	*x*_14_
Aperture	*x*_15_
MeJA	*x*_16_

## Results and discussion

Upon analyzing all the models, M16 showed the most consistent result. Unlike M16, the other 15 models did not show the diminished stomatal closure level when guard cells were presented with combined ABA and ET stimulus. The model considered the logical operator on AXO_2_ and delay mechanism on the antioxidants. Moreover, the model incorporated the hypothesized connection between pH and ET.

By examining M16 closely, we deduced that in the absence of both ET and ABA, guard cells cannot sustain stomatal closure. This result was expected since both ET and ABA are effectors of stomatal closure response [2,26]. Moreover, the results confirmed the role of ABA and ET in inducing closure of the stomata. The increase in the level of ABA was important in maintaining the closure. Closure was sustained when an ABA level of 0.35 was introduced. Similar results were observed when ET was introduced in the system.

**Fig 3:**
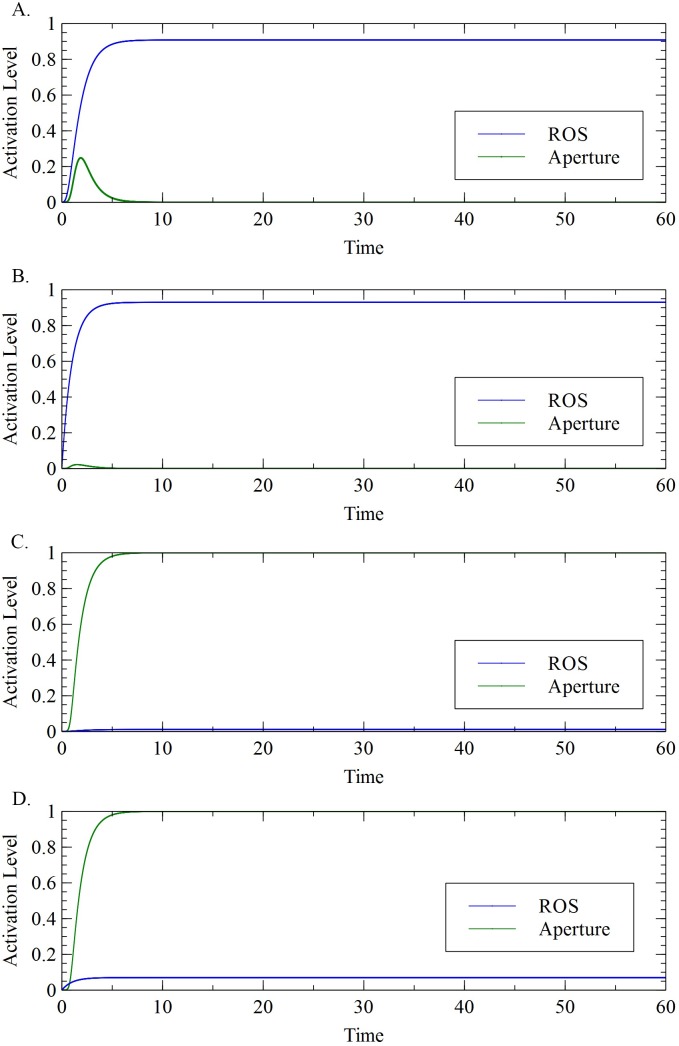
Activation level of ROS and aperture. (A) ABA or ET level is equal to 1; (B) MeJA level equal to 1; (C) ABA or ET level is equal to 0.2; (D) MeJA level is equal to 0.2.

Results also showed that a sufficient level of ROS production was needed to maintain the closed state of stomata (Fig 3). This confirmed the significance of ROS in ABA and ET-induced stomatal closure [2,18,24,27,28]. When the level of ROS is close to zero, the stomata were open.

**Fig 4:**
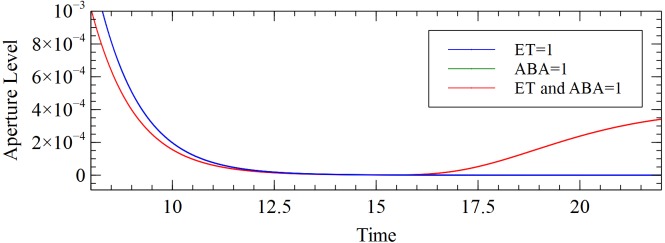
Aperture response to combined ABA and ET stimulus.

Under the combined ABA and ET stimulus, stomatal closure is diminished as compared to the effect of either individual ABA or ET (Fig 4). This is consistent with the reports in [2,18,29]. The low level of ROS, due to the antioxidant response that activates when both ABA and ET are present, corresponds to the diminished closure. The model also confirmed the important role of the two antioxidant mechanisms in the stomatal closure. These mechanisms were the delayed response activated by a single stimulus and the more rapid antioxidant activity that is only activated when both ABA and ET stimuli are present.

**Fig 5:**
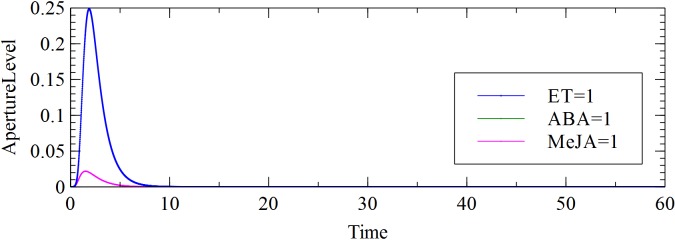
Aperture response to ABA, ET and MeJA.

The model was extended by establishing a crosstalk with another phytohormone, MeJA. Two connections were added: activation of ROS by MeJA and activation of NO by MeJA. Observation and analysis show that MeJA had a positive effect on stomatal closure, as shown in Fig 5. The MeJA-induced stomatal closure was caused by the increased level of both NO and ROS, both of which play a significant role in the guard cell MeJA signalling [9]. When compared to the individual effect of ABA or ET, MeJA was slightly inducing more closure than ABA or ET.

**Fig 6:**
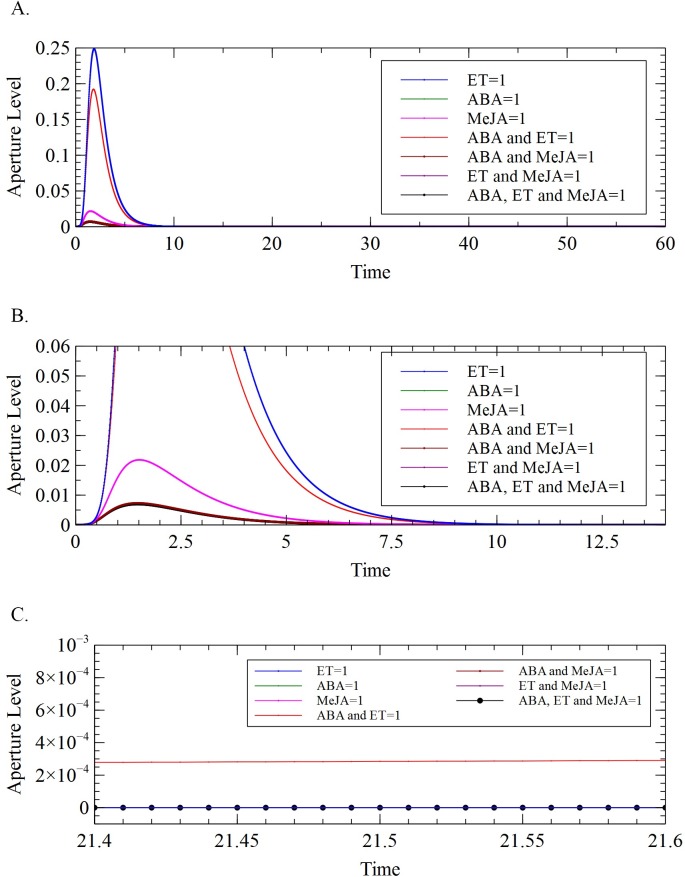
(A) Aperture levels of stomata at different levels of ABA, ET, MeJA and their combinations (B)-(C) magnified versions of (A) at different time intervals.

The interactions of ET with MeJA and of ABA with MeJA were also investigated using the model. Results showed that MeJA enhanced the effect of both ABA and ET in the closing of the stomata. The enhanced effect is apparent since MeJA directly evokes ROS and NO productions that promote closure. However, a significant decrease on the level of stomatal closure was observed when the system was subjected to the combined stimulus of the three hormones (Fig 6). This may be caused by the activation of the antioxidant AXO_2_ that reverses stomatal closure when both ABA and ET are present.

## Summary, conclusion and recommendation

In this study, we constructed a mathematical model of guard cell transduction network for stomatal closure involving the phytohormones ABA, ET and MeJA using continuous logical modelling framework. Based on this, we verified the existing findings about the role of antioxidants in the closure mechanism, as well as the diminished closure level of stomata with combined ABA-ET stimulus. A significant part of our research was the analysis of the ABA-ET-MeJA crosstalk.

Sixteen models were constructed to analyze the signalling network. Results showed that M16 exhibited the most consistent outcome. It considered the logical operator on AXO_2_ and the hypothesized connection between pH and ET. The model showed that guard cells cannot sustain stomatal closure in the absence of both ET and ABA.

Stomatal closure is diminished under the combined ABA and ET stimulus as compared to the effect of either ABA or ET individually. The low level of ROS due to the response of antioxidant that activates when both ABA and ET are present resulted to diminishing closure.

An extension of the model was established through crosstalk with another phytohormone, MeJA. The activation of ROS by MeJA and activation of NO by MeJA were additional connections in the model. It was shown that MeJA induces stomatal closure. Results showed that MeJA improved the effect of both ABA and ET in stomatal closure. However, when the system was subjected to the combined stimulus of the three hormones, a significant decrease on the level of the closure was observed.

The use of standard methodology proved to be helpful in describing the behavior of components of the guard cell signalling network. Despite its simpler approach, the model was able to present the important characteristics of the system’s dynamics. This is particularly important for when networks become large, it becomes more difficult to build predictive quantitative models. Additionally, mechanistic information and kinetic parameter data are often not fully available for complex networks.

Our analysis only included established interactions of ABA, ET, and MeJA on stomatal closure in plants. Further analysis can be done when newly found pathways are incorporated in the transduction network. Furthermore, the effect of other phytohormones can be examined using the similar modelling framework.

## Supporting information

S1 Appendix Source code for the continuous logical modelling implementation. (DOCX)

## Abbreviations

ABA: abscisic acid
AOX_1_: antioxidant 1
AOX_2_: antioxidant 2
AtrbohF: Arabidopsis thaliana respiratory burst oxidase homolog F
ABI1: ABA-insensitive 1
ET: ethylene
I_K,in_: inwards-rectifying K^+^ channels
I_K,out_: outwards-rectifying K^+^ channels
$K_{in}^{+}$: active I_K,in_
$K_{out}^{+}$: active I_K,out_
MeJA: methyl jasmonate
NIA1: nitrate reductase 1
NO: nitric oxide
ODEs: ordinary differential equations
pH_cyt_: cystolic pH
ROS: reactive oxygen species
