# Supplementary material for: A mathematical model of the interaction of abscisic acid, ethylene and methyl jasmonate on stomatal closure in plants
Source: PLoS One. 2017 Feb 9;12(2):e0171065. doi: 10.1371/journal.pone.0171065 (PMC5300265; doi:10.1371/journal.pone.0171065)
Supplement: S1 Appendix — (DOCX) [file pone.0171065.s001.docx]

**Source Code for the Continuous Logical Modelling Implementation**

METHOD RK4

STARTTIME = 0

STOPTIME=60

DT = 0.01

; Initialization Equations

init x3=0

init x4=0

init x5=0

init x6=0

init x7=0

init x8=0

init x9=0

init x10=0

init x11=0

init x12=0

init x13=0

init x14=0

init x15=0

; Parameter Values

x1=1

x2=0

x16=0

a1=1

a2=1

a3=1

a4=1

a5=1

a7=1

a9=1

a10=1

a11=1

a13=1

a14=1

a16=1

b6=1

b8=1

b11=1

b12=1

g3=1

g4=1

g5=1

g6=1

g7=1

g8=1

g9=1

g10=1

g11=1

g12=1

g13=1

g14=1

g15=1

h=10

;Total Input to the node equations

w3=((1+a1+a2)/(a1+a2))*((a1*x1+a2*x2)/(1+a1*x1+a2*x2))

w4=((1+a1+a2)/(a1+a2))*((a1*x1+a2*x2)/(1+a1*x1+a2*x2))

w5=((1+a1+a2)/(a1+a2))*((a1*x1+a2*x2)/(1+a1*x1+a2*x2))

w6=((1+a3)/(a3))*((a3*x3_delayed)/(1+a3*x3_delayed))

w7=((1+a4+a16)/(a4+a16))*((a4*x4+a16*x16)/(1+a4*x4+a16*x16))*(1-(((1+b6+b8)/(b6+b8))*((b6*x6+b8*x8)/(1+b6*x6+b8*x8))))

w8=((1+a5)/(a5))*((a5*x5_delayed)/(1+a5*x5_delayed))

w9=((1+a1+a2)/(a1+a2))*((a1*x1+a2*x2)/(1+a1*x1+a2*x2))

w10=((1+a7)/(a7))*((a7*x7)/(1+a7*x7))

w10=((1+a7)/(a7))*((a7*x7)/(1+a7*x7))

w11=((1+a10+a16)/(a10+a16))*((a10*x10+a16*x16)/(1+a10*x10+a16*x16))

w12=((1+a9+a11)/(a9+a11))*((a9*x9+a11*x11)/(1+a9*x9+a11*x11))

w13=1-(((1+b11)/(b11)))*((b11*x11)/(1+b11*x11))

w14=((1+a13)/(a13))*((a13*x13)/(1+a13*x13))*(1-(((1+b12)/(b12)))*((b12*x12)/(1+b12*x12)))

w15=((1+a14)/(a14))*((a14*x14)/(1+a14*x14))

;Delay Mechanism

x3_delayed = delay(x3, delay_time)

flow a_real = x3

flow a_delayed = x3_delayed

init a_real = 0

init a_delayed = 0

delay_time = 111

x5_delayed = delay(x5, delay_time1)

flow b_real = x5

flow b_delayed = x5_delayed

init b_real = 0

init b_delayed = 0

delay_time1 = 12

;Associated Differential Equations

d/dt(x3)=(-exp(.5*h)+exp(-h*(w3-.5)))/((1-exp(.5*h))*(1+exp(-h*(w3-.5))))-g3*x3

d/dt(x4)=(-exp(.5*h)+exp(-h*(w4-.5)))/((1-exp(.5*h))*(1+exp(-h*(w4-.5))))-g4*x4

d/dt(x5)=IF (x1>0 AND x2>0) THEN (-exp(.5*h)+exp(-h*(w5-.5)))/((1-exp(.5*h))*(1+exp(-h*(w5-.5))))-g5*x5 ELSE 0

d/dt(x6)=(-exp(.5*h)+exp(-h*(w6-.5)))/((1-exp(.5*h))*(1+exp(-h*(w6-.5))))-g6*x6

d/dt(x7)=(-exp(.5*h)+exp(-h*(w7-.5)))/((1-exp(.5*h))*(1+exp(-h*(w7-.5))))-g7*x7

d/dt(x8)=(-exp(.5*h)+exp(-h*(w8-.5)))/((1-exp(.5*h))*(1+exp(-h*(w8-.5))))-g8*x8

d/dt(x9)=(-exp(.5*h)+exp(-h*(w9-.5)))/((1-exp(.5*h))*(1+exp(-h*(w9-.5))))-g9*x9

d/dt(x10)=(-exp(.5*h)+exp(-h*(w10-.5)))/((1-exp(.5*h))*(1+exp(-h*(w10-.5))))-g10*x10

d/dt(x11)=(-exp(.5*h)+exp(-h*(w11-.5)))/((1-exp(.5*h))*(1+exp(-h*(w11-.5))))-g11*x11

d/dt(x12)=(-exp(.5*h)+exp(-h*(w12-.5)))/((1-exp(.5*h))*(1+exp(-h*(w12-.5))))-g12*x12

d/dt(x13)=(-exp(.5*h)+exp(-h*(w13-.5)))/((1-exp(.5*h))*(1+exp(-h*(w13-.5))))-g13*x13

d/dt(x14)=(-exp(.5*h)+exp(-h*(w14-.5)))/((1-exp(.5*h))*(1+exp(-h*(w14-.5))))-g14*x14

d/dt(x15)=(-exp(.5*h)+exp(-h*(w15-.5)))/((1-exp(.5*h))*(1+exp(-h*(w15-.5))))-g15*x15

;End of program
